# Thin-Slice Magnetic Resonance Imaging-Based Radiomics Signature Predicts Chromosomal 1p/19q Co-deletion Status in Grade II and III Gliomas

**DOI:** 10.3389/fneur.2020.551771

**Published:** 2020-10-22

**Authors:** Ziren Kong, Chendan Jiang, Yiwei Zhang, Sirui Liu, Delin Liu, Zeyu Liu, Wenlin Chen, Penghao Liu, Tianrui Yang, Yuelei Lyu, Dachun Zhao, Hui You, Yu Wang, Wenbin Ma, Feng Feng

**Affiliations:** ^1^Department of Neurosurgery, Peking Union Medical College Hospital, Chinese Academy of Medical Sciences and Peking Union Medical College, Beijing, China; ^2^Department of Radiology, Peking Union Medical College Hospital, Chinese Academy of Medical Sciences and Peking Union Medical College, Beijing, China; ^3^Department of Radiology, Beijing Chao-Yang Hospital, Capital Medical University, Beijing, China; ^4^Department of Pathology, Peking Union Medical College Hospital, Chinese Academy of Medical Sciences and Peking Union Medical College, Beijing, China

**Keywords:** MRI, radiomic, glioma, chromosomal 1p/19q co-deletion, spacing

## Abstract

**Objective:** Chromosomal 1p/19q co-deletion is recognized as a diagnostic, prognostic, and predictive biomarker in lower grade glioma (LGG). This study aims to construct a radiomics signature to non-invasively predict the 1p/19q co-deletion status in LGG.

**Methods:** Ninety-six patients with pathology-confirmed LGG were retrospectively included and randomly assigned into training (*n* = 78) and validation (*n* = 18) dataset. Three-dimensional contrast-enhanced T1 (3D-CE-T1)-weighted magnetic resonance (MR) images and T2-weighted MR images were acquired, and simulated-conventional contrast-enhanced T1 (SC-CE-T1)-weighted images were generated. One hundred and seven shape, first-order, and texture radiomics features were extracted from each imaging modality and selected using the least absolute shrinkage and selection operator on the training dataset. A 3D-radiomics signature based on 3D-CE-T1 and T2-weighted features and a simulated-conventional (SC) radiomics signature based on SC-CE-T1 and T2-weighted features were established using random forest. The radiomics signatures were validated independently and evaluated using receiver operating characteristic (ROC) curves. Tumors with IDH mutations were also separately assessed.

**Results:** Four radiomics features were selected to construct the 3D-radiomics signature and displayed accuracies of 0.897 and 0.833, areas under the ROC curves (AUCs) of 0.940 and 0.889 in the training and validation datasets, respectively. The SC-radiomics signature was constructed with 4 features, but the AUC values were lower than that of the 3D signature. In the IDH-mutated subgroup, the 3D-radiomics signature presented AUCs of 0.950–1.000.

**Conclusions:** The MRI-based radiomics signature can differentiate 1p/19q co-deletion status in LGG with or without predetermined IDH status. 3D-CE-T1-weighted radiomics features are more favorable than SC-CE-T1-weighted features in the establishment of radiomics signatures.

## Introduction

Glioma remains the most lethal central nervous system (CNS) tumor despite advances in therapeutic approaches, including surgical resection, radiotherapy, and chemotherapy (1). The genomic analysis of diffuse lower grade glioma (LGG; refers to World Health Organization [WHO] grade II and III glioma) has identified several important molecular biomarkers for subgroup identification to guide clinical decisions in addition to histopathological classification (2). The co-deletion of chromosome arms 1p and 19q, which is mediated by 1;19 translocation (3), was strongly correlated with a oligodendroglial origin and is a biologically discrete subtype that harbors the mutation of CIC and FUBP1 as well as the activation of TERT (4). LGG patients with 1p/19q co-deletion have a significantly better prognosis than 1p/19q intact patients regardless of the therapeutic regimen and display higher sensitivity to the procarbazine, lomustine, and vincristine (PCV) regimen (5–7). Therefore, the status of 1p/19q has been recognized as a diagnostic, prognostic, and predictive biomarker in LGG (8). However, the 1p/19q status is mostly measured by fluorescence *in situ* hybridization (FISH), polymerase chain reaction (PCR)-based microsatellite analysis, or GeneChip-based copy number array (9) based on tumor tissue, which is restricted by the unattainability of tumor samples, tumor heterogeneity, relatively long detection periods, and the high level of training required. In addition, only patients with 1p/19q intact tumors but not those with 1p/19q co-deleted tumors display a significant survival benefit from gross total resection (GTR) compared to subtotal resection (STR) (10, 11), highlighting the importance of acquiring the 1p/19q status prior to surgery. Therefore, a non-invasive evaluation of the 1p/19q status is intensively needed to facilitate the personalized treatment of glioma patients.

Although magnetic resonance imaging (MRI) is continuously employed in the evaluation of CNS diseases, the differences in tumor location, tumor border, contrast enhancement, diffusion, and perfusion patterns between 1p/19q co-deleted and 1p/19q intact LGGs were non-significant (12, 13). The T2 fluid-attenuated inversion recovery (T2-FLAIR) mismatch sign has been recently recognized as a characteristic for 1p/19q intact tumors with high specificity but low sensitivity (14), and further imaging characteristics need to be identified to distinguish the 1p/19q status. Radiomics, a recently emerging technique, can extract high-throughput radiomics features from imaging data to quantitatively describe tumor characteristics and build a mathematical model to predict tumor phenotype through relevant radiomics features (15). Several MRI-based radiomics studies of glioma have investigated the association between selected radiomics features and WHO grading, molecular characteristics, clinical manifestations, and patient prognosis (16–18). A few studies have involved the non-invasive prediction of 1p/19q status through a radiomics approach but display only moderate prediction value (18–26), and further investigation is needed to establish a reliable radiomics signature. In addition, previous studies were conducted using MR images acquired with diverse spacing (ranging from 1 to 5–6 mm for contrast-enhanced T1 [CE-T1]-weighted images), and whether such differences would influence the performance of the prediction model remains to be explored.

This study retrospectively investigates pretreatment LGG MRI characteristics through a radiomics approach, aiming to build a reliable signature to non-invasively predict the 1p/19q co-deletion status in LGG patients.

## Materials and Methods

### Patients

This study retrospectively included pathologically confirmed LGG patients diagnosed at Peking Union Medical College Hospital (PUMCH) from August 2010 to October 2019. Patients were included if the following criteria were met: (1) adults with histopathologically diagnosed with WHO grade II or grade III oligodendroglioma, astrocytoma, or oligoastrocytoma; (2) adequate paraffin-embedded tumor tissue for the detection of IDH and 1p/19q status; (3) preoperative three-dimensional contrast-enhanced T1 (3D-CE-T1)-weighted and T2-weighted MRI; (4) no significant central necrosis or cyst inside the tumor region (27, 28); (5) no previous history of CNS tumors; and (6) no radiation or anticancer drugs (e.g., chemotherapy) delivered prior to MRI and surgery. The study design was approved by the Institutional Review Board, and all patients provided informed consent. Finally, 96 local patients met the inclusion criteria and were randomly assigned to the training dataset (*n* = 78) and validation dataset (*n* = 18).

### 1p/19q FISH Examination and IDH Mutation Detection

The 1p/19q co-deletion status was determined by FISH as described by Snuderl et al. (29). Formalin-fixed paraffin-embedded tumor slides were prepared, and hematoxylin-eosin (HE) staining was performed to identify the tumor area. Two separate FISH were performed using SPEC Dual Color Probe (Zytovision, Germany), with chromosome 1p36 labeled orange and 1q25 labeled green on one slide, and chromosome 19p13 labeled green and 19q13 labeled orange on the other slide. Nuclei were stained with 4′,6-diamidino-2-phenylindole (DAPI), and fluorescent signals of 100 non-overlapping nuclei within the tumor area were measured using a BX41 TRF fluorescence microscope (Olympus, Japan). Glioma was defined as “1p (or 19q) loss” if the ratio of 1p: 1q (or 19q: 19p) was <0.75; otherwise, the tumor was recognized as “1p (or 19q) intact.”

IDH1 and IDH 2 mutations were detected by direct sequencing as described by Horbinski et al. (30). In brief, DNA was extracted from formalin-fixed paraffin-embedded tumor tissue using a Simlex OUP® FFPE DNA extraction kit (TIB, China), and subsequent PCR was performed using a Verity 96-Well Thermal Cycler (ThermoFisher, US) to amplify DNA fragments that contain IDH mutation hotspots (IDH1 R132 and IDH2 R172). The PCR products were then purified and sequenced using the Genetic Analyzer 3500 (ThermoFisher, US).

### MRI Data Acquisition and Tumor Segmentation

Preoperative MRI examinations were performed on a 3.0-T MRI scanner (Discovery MR750, GE, US). 3D-CE-T1-weighted images (gadolinium chelate, 0.1 mmol/kg; slice thickness 1 mm; slice interval 1 mm; repetition time, 6.2–8.3 ms; echo time, 2.6–3.2 ms; inversion time, 400 ms) and T2-weighted images (slice thickness 5–6 mm; slice interval 6 mm; repetition time, 3,440–4,060 ms; echo time, 85–103 ms) were obtained. All MR images were acquired with a matrix size of 256 × 256 and interpolated into a size of 512 × 512 as a standard protocol for clinical use. The original DICOM data were converted to NIfTI format for later processing and anonymity.

T2-weighted images were coregistered to 3D-CE-T1-weighted images for a clear delineation of the tumor boundary as well as the elimination of head movement. The three-dimensional region of interest (ROI), which is consistent with the area for GTR (abnormal CE-T1 areas for contrast-enhanced tumors and abnormal T2 areas for non-contrast-enhanced tumors) (31, 32), was manually delimitated by two neurosurgeons using ITK-SNAP software (http://www.itksnap.org/pmwiki/pmwiki.php) on 3D-CE-T1-weighted images. The ROI was subsequently coregistered to T2-weighted images using FSL (Functional Magnetic Resonance Imaging of the Brain Analysis Group, Oxford University) (33–35) to prohibit inconsistencies in the ROI between different sequences. The ROI was then evaluated by a senior neuroradiologist. If the difference between ROIs was ≤ 5% for the two neurosurgeons, the final ROIs were defined by the overlapping area of the initial ROIs; if the difference between ROIs was >5%, the neuroradiologist made the final decision.

### Down-Sampling of 3D-CE-T1-Weighted Images

**Figure 1:**
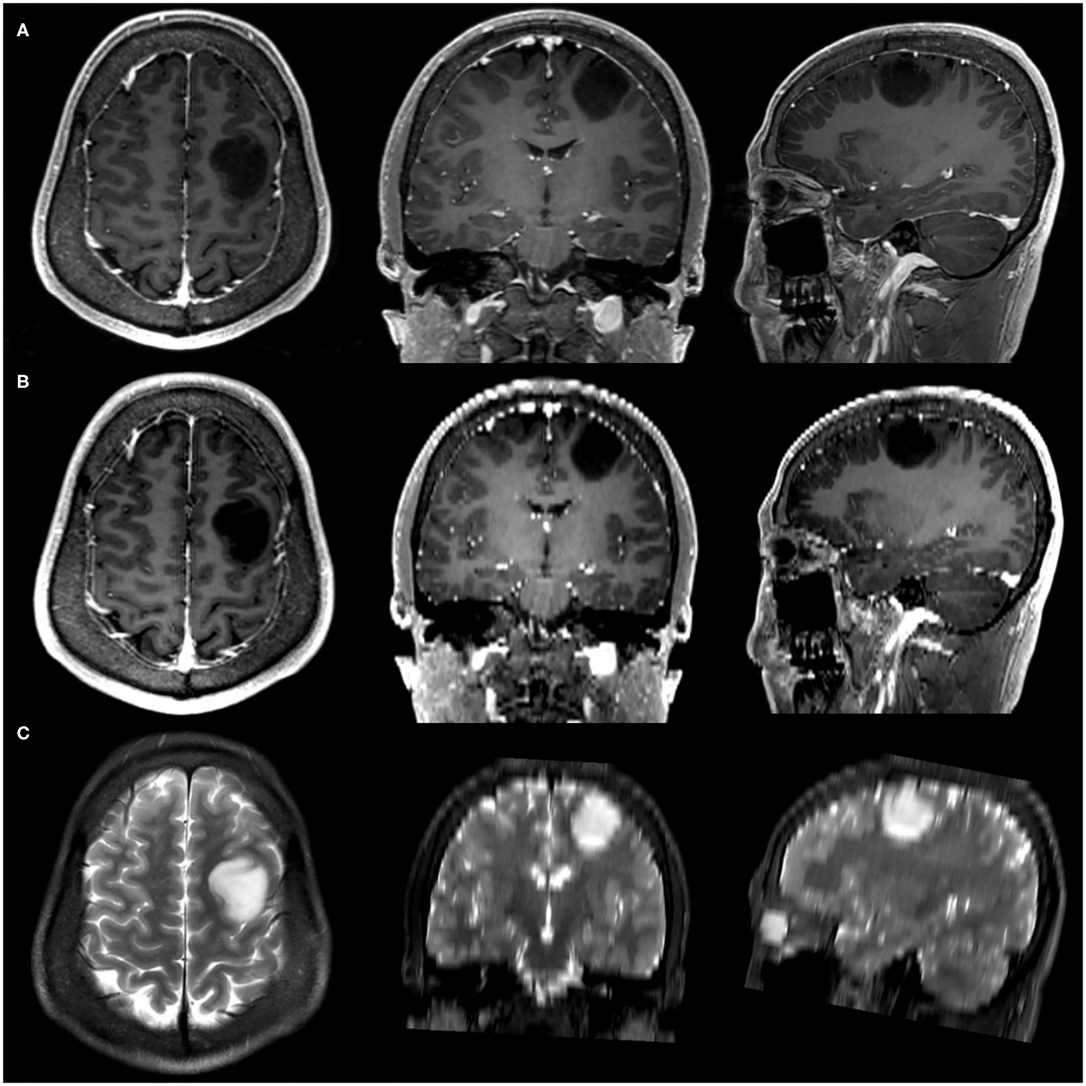
Example of three-dimensional contrast-enhanced T1 (3D-CE-T1)-weighted **(A)**, simulated-conventional contrast-enhanced T1 (SC-CE-T1)-weighted **(B)**, and T2-weighted **(C)** images.

Simulated-conventional contrast-enhanced T1 (SC-CE-T1)-weighted images were down-sampled from 3D-CE-T1-weighted images to investigate the impact of acquisition spacing on the performance of radiomics signatures. One of every five slices was retained in the interpolated 0.5 mm-thick 3D-CE-T1 series, which created a SC-CE-T1 series with a 0.5 mm slice thickness and 2.5 mm slice interval to replicate the slice interval of conventional CE-T1 images without causing significant blurring or a partial volume effect. The spatial location and spacing information was updated accordingly. Examples of original 3D-CE-T1-weighted and T2-weighted images as well as the down-sampled SC-CE-T1-weighted images are shown in Figure 1.

### Radiomics Feature Extraction, Selection, and Radiomics Signature Construction

The brightness of the 3D-CE-T1, SC-CE-T1, and T2-weighted images was normalized by centering the voxels at the mean value with standard deviation (SD) on the basis of all gray values using the preset module of PyRadiomics (2.1.0, http://www.radiomics.io/) (36). A total of 107 radiomics features were extracted from the ROI of each imaging modality with PyRadiomics (36). All radiomics features were scaled based on the SD of the training dataset to avoid data fluctuation, and the scale was subsequently applied in the validation dataset. The extracted features are summarized in Table 1 and detailed in Supplementary Material 1.

**Table 1:** Summary of the extracted radiomics features for each imaging modality.

**Feature classes**	**Numbers**	**Notes**
Shape	14	Quantifies the characteristics of tumor's shape.
First Order	18	Reflects the voxel-alone based statistical values.
Texture	75	Describes surface texture.
GLCM	24	Exhibits the relationship of adjacent voxels.
GLRLM	16	Manifests the consecutive voxels with same gray level values.
GLSZM	16	Reveals gray level zones.
GLDM	14	Shows the gray level dependencies.
NTGDM	5	Presents the difference between a certain gray value and the average gray value of its neighbors within a specific distance.
Total	107	

*GLCM, Gray level co-occurrence matrix; GLRLM, Gray level run length matrix; GLSZM, Gray level size zone matrix; GLDM, Gray level dependence matrix; NTGDM, Neighboring gray tone difference matrix*.

Radiomics features were selected by the least absolute shrinkage and selection operator (LASSO) in the training dataset with code constructed using Scikit-learn (v0.20.0, http://scikit-learn.org) (37). A 3D-radiomics signature and a simulated conventional (SC)-radiomics signature were constructed based on random forest, and feature reduction was also allowed at this step. The 3D-radiomics signature was established by concatenating 3D-CE-T1- and T2-weighted features together and performing *de novo* selection. Similarly, the SC-radiomics signature was built from the radiomics features of SC-CE-T1- and T2-weighted images.

### Signature Validation and Comparison

The radiomics signatures were validated independently in the validation dataset. Accuracy, sensitivity, specificity, and area under the receiver operating characteristic (ROC) curve (AUC) were calculated to evaluate the performance of the signatures. The DeLong test was utilized to calculate the difference between ROC curves as well as the confidence interval of the AUC values. The radiomics signature that had the best prediction performance was selected to non-invasively differentiate the 1p/19q status in LGG.

### Subgroup Analysis

Since IDH-mutated LGGs have a significantly diverse pathogenesis and better prognosis than IDH-wild type LGGs (4) and the 1p/19q co-deletion status is diagnostic of oligodendroglioma origin when coexisting with IDH mutation (38), the selected radiomics signature was further evaluated in IDH-mutated LGGs in both the training and validation datasets to explore whether the signature can predict the 1p/19q status in specifically IDH-mutated LGGs. The selected radiomics signature was also assessed in WHO grade II tumors and WHO grade III tumors separately.

### Occlusion Map Generation

Occlusion map can imply the contribution of a certain portion of a medical image to a computational value (i.e., in our research, radiomics features) by placing a black square mask over the image and comparing the altered computational values with the original ones (39). It is a comprehensive computational method to investigate regional contribution to certain continuous variable, despite its high cost on computing power and limits on some algorithms. A higher value in the occlusion map indicates a greater contribution of the veiled portion. A 5 × 5 voxel square mask moving at a suitable distance of 5 voxels was utilized to generate the occlusion map of the selected radiomics features. These moving distances were selected after balancing the computation time and resolution of the output image.

### Statistical Analysis

Statistical analysis was performed with R 3.6.2 (https://www.r-project.org) and Python 3.6.5 (https://www.python.org).

## Results

### Patient Characteristics

The baseline characteristics of the 96 included patients are summarized in Table 2. No significant differences in sex, age, or WHO grade were observed between the training (*n* = 78) and validation datasets (*n* = 18) (*p* = 0.200–0.864). There were 16 (20.5%) and 6 (33.3%) patients with 1p/19q co-deletion in the training and validation datasets, respectively, and the differences in the distribution of 1p/19q status were non-significant (*p* = 0.392). In addition, the IDH mutation status was also balanced between the training and validation datasets (*p* = 0.918), and in accordance with the 2016 WHO classification of CNS tumors, all 1p/19q co-deleted tumors harbored IDH1 or IDH2 mutations.

**Table 2:** Baseline characteristics of the training and validation dataset.

**Characteristics**	**Training**	**Validation**	***P*-value**
	**dataset**	**dataset**	
Sex			0.293
Male	50 (64.1%)	9 (50.0%)	
Female	28 (35.9%)	9 (50.0%)	
Age (Mean ± SD)	45.8 ± 11.4	44.4 ± 15.2	0.864
1p19q status			0.392
Co-deletion	16 (20.5%)	6 (33.3%)	
Intact	62 (79.5%)	12 (66.7%)	
IDH status			0.918
IDH mutation	51 (65.4%)	12 (66.7%)	
IDH wildtype	27 (34.6%)	6 (33.3%)	
WHO Grade			0.200
Grade II	44 (56.4%)	7 (38.9%)	
Grade III	34 (43.6%)	11 (61.1%)	

*Chi-square test, Fisher's exact test or independent sample t-test, as appropriate, were utilized to calculate the statistical differences. Unless otherwise noted, data in the table described the number and percentages of patients*.

### Feature Selection

**Figure 2:**
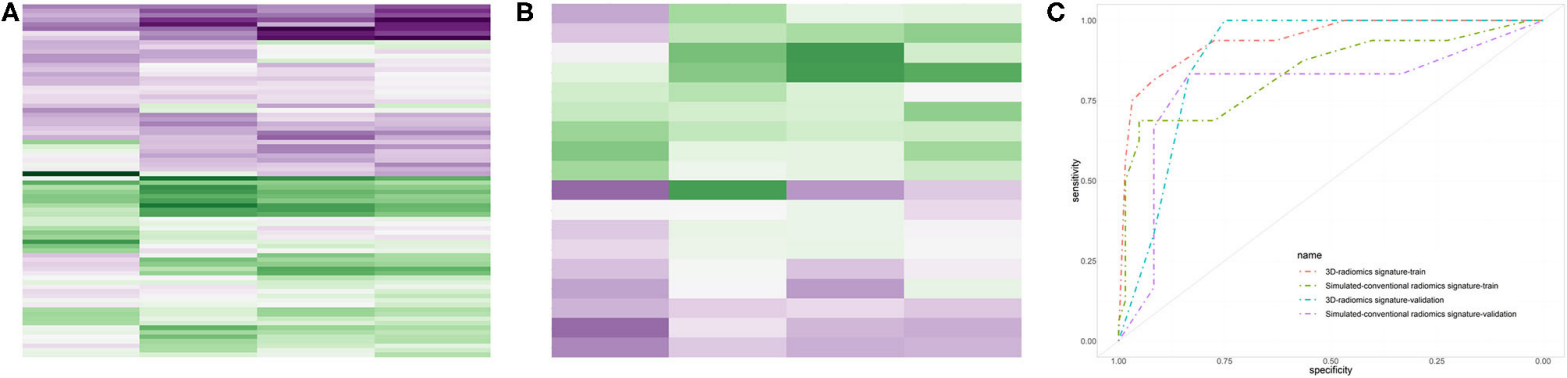
Performances of the radiomics signatures. The heatmaps of the selected features in the 3D-radiomics signature in the training dataset **(A)** and validation dataset **(B)** were clustered by 1p/19q co-deletion status. The receiver operating characteristic (ROC) curves of the 3D- and SC-radiomics signatures **(C)** are also shown.

Five radiomics features were selected by LASSO algorithm, and four radiomics features, three of which originated from 3D-CE-T1-weighted images and one of which came from T2-weighted images, were selected to build the 3D-radiomics signature after one feature excluded by random forest. The selected features are detailed in Table 3. Clustered heatmaps of the selected features in the training and validation datasets are displayed in Figures 2A,B. Likewise, four final radiomics features (one SC-CE-T1-weighted and three T2-weighted features) were selected to construct the SC-radiomics signature. These features are detailed in Supplementary Material 2.

**Table 3:** The selected features in the 3D-radiomics signature.

**Feature name**	**Modality**	**Matrix**
Informational Measure of Correlation 2	3D-CE-T1-weighted	GLCM
Correlation	3D-CE-T1-weighted	GLCM
Dependence Entropy	3D-CE-T1-weighted	GLDM
Major Axis Length	T2-weighted	Shape

*3D-CE-T1-weighted, three-dimensional contrast enhanced T1-weighted; GLCM, Gray level co-occurrence matrix; GLDM, Gray level dependence matrix*.

### Signature Construction and Comparison

The 3D-radiomics signature was constructed with 4 final features and displayed an accuracy of 0.897 and AUC of 0.940 (0.877–1.000) in the training dataset, and an accuracy of 0.833 and AUC of 0.889 (0.735–1.000) in the validation dataset. The sensitivity and specificity were relatively balanced. However, the performance of the SC-radiomics signature was lower than that of the 3D signature, with accuracies similar to that of the 3D-radiomics signature but AUCs of 0.838 (0.710–0.966) and 0.792 (0.514–1.000) in the training and validation datasets, respectively. The DeLong test was employed and displayed a trend of differences between the two signatures in the training (*p* = 0.158) and validation (*p* = 0.518) datasets. The 3D-radiomics signature was selected for the non-invasive prediction of 1p/19q status in LGGs due to its better performance. The ROC curves of the radiomics signatures in the training and validation datasets are displayed in Figure 2C. The performance metrics of the 3D-radiomics signature and SC-radiomics signature are shown in Table 4. Bar charts of the 3D-radiomics signature are exhibited in Supplementary Material 3.

**Table 4:** Prediction performance of the 3D-radiomics signature and sc-radiomics signature in the whole population, and the performance of 3D- radiomics signature in IDH-mutated subgroup.

**Prediction model**	**Training dataset**				**Validation dataset**			
	**ACC**	**SEN**	**SPE**	**AUC (95% CI)**	**ACC**	**SEN**	**SPE**	**AUC (95% CI)**
3D-radiomics signature	0.897	0.813	0.919	0.940 (0.877–1.000)	0.833	1.000	0.750	0.889 (0.735–1.000)
SC-radiomics signature	0.897	0.688	0.952	0.838 (0.710–0.966)	0.833	0.833	0.833	0.792 (0.514–1.000)
3D-radiomics signature on IDH-mutated subgroup	0.920	0.800	0.971	0.950 (0.886–1.000)	1.000	1.000	1.000	1.000 (1.000–1.000)
3D-radiomics signature on WHO grade II subgroup	0.909	0.889	0.914	0.937 (0.843–1.000)	1.000	1.000	1.000	1.000 (1.000–1.000)
3D-radiomics signature on WHO grade III subgroup	0.882	0.714	0.926	0.939 (0.862–1.000)	0.727	1.000	0.571	0.750 (0.444–1.000)

*CI of ROC curve with AUC = 1 is always 1-1 which may be misleading. 3D, three-dimensional; SC, simulated-conventional; ACC, accuracy; SEN, sensitivity; SPE, specificity; AUC, area under curve; CI, confidence interval*.

### Subgroup Analysis

The performance of the selected radiomics signature was also evaluated among IDH-mutated tumors, WHO grade II tumors, and WHO grade III tumors. The 3D-radiomics signature displayed an accuracy of 0.920 and AUC of 0.950 (0.886–1.000) in the IDH-mutated subset of the training dataset; meanwhile, all IDH-mutated samples in the validation dataset were correctly predicted. The performance of the 3D-radiomics signature in WHO grade II tumors and WHO grade III tumors were also relatively balanced. The performance metrics of the subgroup analysis are detailed in Table 4.

## Discussion

In our study, first-order and texture features from the 3D-CE-T1-weighted, SC-CE-T1-weighted, and T2-weighted images were extracted and selected. A 3D-radiomics signature based on the 3D-CE-T1 and T2-weighted features, and a SC-radiomics signature based on the SC-CE-T1 and T2-weighted features, were established to predict the 1p/19q co-deletion status. The 3D-radiomics signature displayed a reliable performance, with accuracies of 0.897 and 0.833, and AUCs of 0.940 and 0.889 in the training and validation datasets, respectively, indicating the capability of the conventional-MRI-based radiomics signature to non-invasively predict the 1p/19q co-deletion status. The 3D signature also outperformed the SC signature, which revealed the advantage of thinner slice images in radiomics studies. In addition, the performance of the 3D-radiomics signature was further validated in IDH-mutated LGGs, suggesting the generalizability of our signature regardless of the predetermined IDH status.

In addition to the well-established diagnostic, prognostic, and predictive values (8), chromosomal 1p/19q co-deletion status also aided the surgical planning of LGGs. Ding et al. (10) suggested that only the 1p/19q intact LGGs but not the 1p/19q co-deleted LGGs would benefit from GTR compared with STR both in the multi-centric local cohort and in the Cancer Genome Atlas (TCGA) validation cohort, and Kawaguchi et al. also reported a non-significant difference of survival between GTR and non-GTR in 1p/19q co-deleted grade III gliomas but a significant difference in 1p/19q intact grade III tumors (11). Thus, the non-invasive measurement of 1p/19q status would better support the pre-operative and intra-operative decision. Previously, the MRI-based T2-FLAIR mismatch sign was recognized as a visual-based imaging characteristic with good interobserver correlation that may determine the 1p/19q status in LGG. A recent systematic review suggested that the T2-FLAIR mismatch sign displayed a sensitivity and specificity of 30 and 73% in determining IDH mutated with 1p/19q co-deleted tumors, and 34 and 98% for IDH mutated with 1p/19q intact tumors (40). However, such imaging characteristics may be limited in clinical application due to the unbalanced sensitivity and specificity, and high-throughput quantitative features are intensively needed to better illustrate the radiological divergences and further predict the 1p/19q status non-invasively. Previous radiomics studies using conventional MRI or advanced MRI sequences to predict 1p/19q status reached AUCs ranging from 0.68 to 0.96 (if reported, without distinguishing the training and validation dataset) (18–26), and our study displayed a competent result, with AUCs around 0.90 for the whole population and further elevated in IDH-mutated tumors, suggesting the capability of our signature for non-invasive 1p/19q detection. In addition, the 3D signature also displayed a balanced sensitivity and specificity, which compensated for the disequilibrium of visual characteristics. Wavelet radiomics features that were based on frequency transformation and wavelet filtering, on the other hand, have restricted visual significance to radiologists and were not included in the current study. It is worth noting that the 1p/19q status can be measured by diverse methodologies, including FISH, GeneChip-based copy number array, or PCR-based microsatellite analysis in clinical practice and displays good concordance with the test results. FISH remains the most widely applicable technique with direct morphology evaluation of chromosomal abnormalities, yet it may be unable to detect the small intragenic variation or partial deletion of the hybridized region due to the relatively large size of the FISH probe (9).

**Figure 3:**
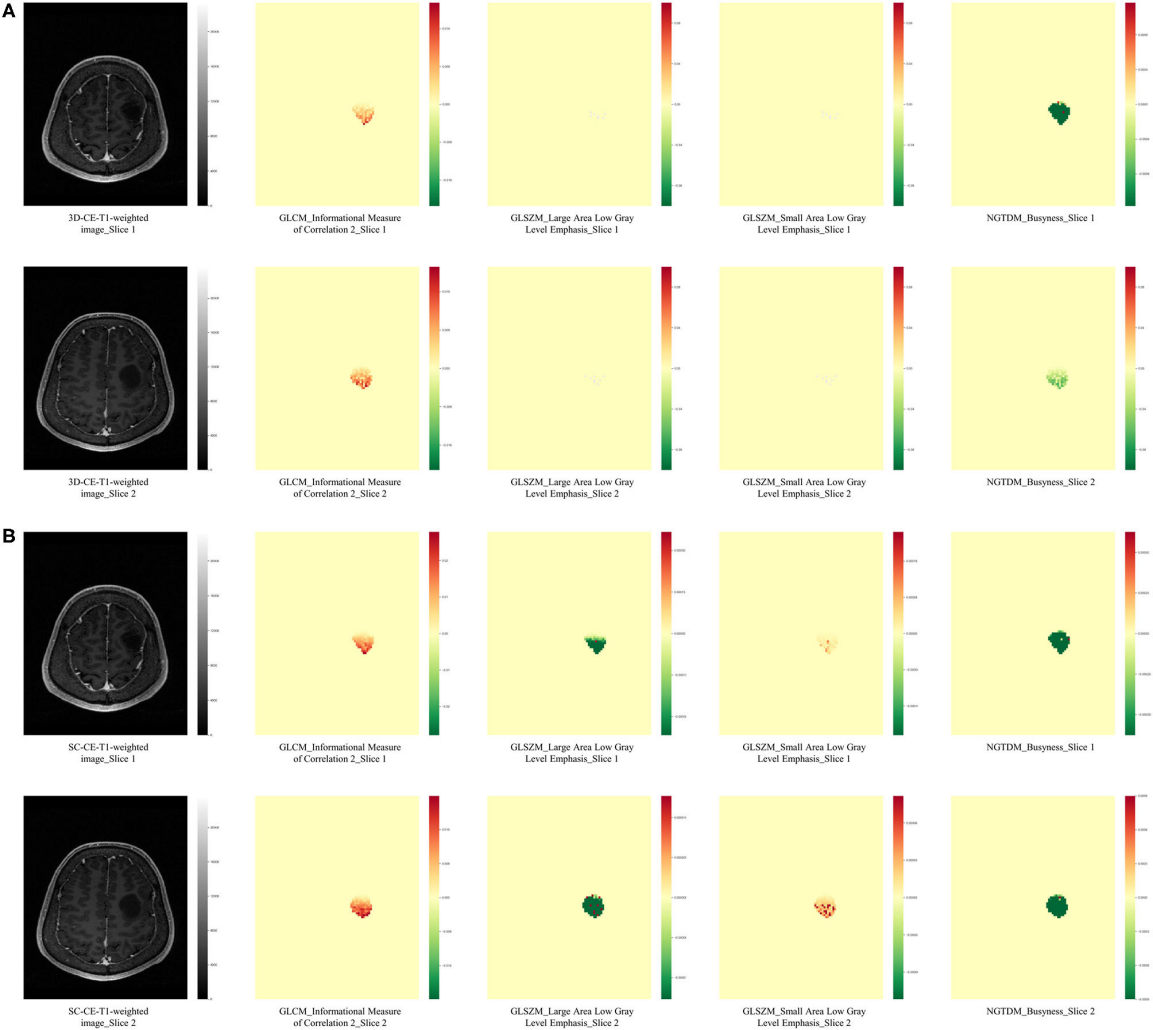
Occlusion maps displayed the influence of spacing on radiomics features. The occlusion maps of four radiomics features from 3D-CE-T1-weighted **(A)** and SC-CE-T1-weighted images **(B)** are presented. As expected, the original axial images (slice 1 and slice 2) from 3D and SC images have a same resolution, while the calculation of radiomics features was influenced. The occlusion maps of the GLCM feature from the SC image were smoother than the feature from the 3D image, probably because a larger number of voxels was included when calculating the texture matrices. The two GLSZM features had minimum differences in the 3D-CE-T1-weighted image-derived occlusion maps, while they were significantly different in the SC-derived maps, indicating the effect of spacing on the calculated value. The NGTDM occlusion map from slice 1 was similar to slice 2 in SC-derived features but different to slice 2 in 3D-derived features, suggesting a location impact of re-sampling to calculated values. GLCM, gray level co-occurrence matrix; GLRLM, gray level run length matrix; NGTDM, neighboring gray tone difference matrix.

Radiomics features are generally sensitive to the spatial resolution of MR images (41), but the influence of acquisition spacing on radiomics features as well as the performance of radiomics signatures remain to be investigated. When evaluating gliomas, MR images (especially CE-T1-weighted images) are usually acquired with slice thicknesses and slice intervals ranging from 1 to 5 mm. Smaller spacing are more conducive to the wide application of neuro-navigation and the need for meticulous presurgical planning, yet larger spacing are more easily acquired across institutes and can facilitate a fast initial evaluation of diseases while also lowering the requirements for computation in radiomics studies. During the training of radiomics models, images from the same modality need to be re-sampled according to the data with the largest spacing, which may result in a loss of information from the small-spacing data. If the small-spacing-based signature has a better performance than the large-spacing-based signature, a thinner image should be routinely acquired and promoted in radiomics studies so that detailed information can be reflected. In our study, a down-sampling of the uniform thin-slice CE-T1-weighted images was performed and generated SC-CE-T1-weighted images that emulate the thick-slice images. This strategy was utilized to expand the slice interval without significantly decreasing the spatial resolution, since averaging the sequential thin slices to stimulate the thick-slice images would introduce blurring and partial volume effects (42). The 3D-radiomics signature performed better than the SC signature with same size of feature set, indicating that the additional imaging details in thin-cut images did contribute to a better performance. In addition, 3 of the 4 features from the 3D-radiomics signature originated from 3D-CE-T1-weighted images, while only 1 of the 4 features from the SC-radiomics signature was derived from the SC-CE-T1-weighted images, which also suggested a higher weight of the 3D image-derived features in the prediction models. The occlusion maps (Figure 3) provide some hint of the changes after resampling. The most obvious change was the dramatically blurred resolution in the direction of the skipping, and some features showed obvious value alterations. When backtracking the mathematical definition of the texture matrices, it is clear that gray level co-occurrence matrix (GLCM), generated with adjacent voxels, will suffer most from enlarged spacing; gray level dependence matrix (GLDM), generated from adjacent voxels at a given distance (usually smaller than the spacing of the SC series), will also undergo a remarkable change. This may explain why the GLCM and GLDM features of the CE-T1-weighted images were selected to establish the 3D-radiomics signature but were neglected in the SC signature. Admittedly, the ideal comparable signatures should be derived from *de novo* thin- and thick-slice sequences, but this is unattainable because of the retrospective nature of our study, and further prospective studies may better illustrate the influence of interslice spacing on radiomics studies.

In addition to chromosomal 1p/19q co-deletion status, IDH1/2 mutation status has also been recognized as a vital diagnostic, prognostic, and predictive biomarker for gliomas (8). LGGs with IDH mutations and 1p/19q co-deletion were classified as molecular pathological oligodendrogliomas, while LGGs with mutated IDH and intact 1p/19q or wild-type IDH were considered to have an astrocytoma origin (38). Thus, we validated our result in IDH-mutated LGGs and displayed AUCs of 0.950–1.000, suggesting the capability of our prediction model to predict 1p/19q status in all LGG patients and in IDH-mutated LGG cohorts. However, the sample size of the IDH-mutated subgroup in the validation dataset was relatively limited, which makes the performance of this group less convincing. Considering the non-invasive nature of the current study, IDH mutation status was not integrated as an inclusion criterion since the non-invasive method for IDH detection may not be sophisticated. Further large cohort radiomics studies to distinguish multi-molecular markers may be necessary to facilitate the preoperative classification of gliomas.

Although the appearance of 1p deletion and 19q deletion are strongly correlated in diffuse gliomas and have been recognized as diagnostic criteria for oligodendroglioma, single 1p loss (with 19q intact) or single 19q loss (with 1p intact) can also occur less frequently with certain clinical meaning (43). Ichimura et al. (44) reported that patients with astrocytoma with total 1p loss had a significantly better prognosis than those with other types of 1p status, and Otani et al. (45) found that patients IDH-mutated astrocytoma with 19q loss also had longer survival times than those with 19q intact astrocytoma, suggesting that single 1p or 19q loss may increase the clinical similarity of the tumor with the 1p/19q co-deleted tumor. In our dataset, 1p-loss/19q-retain was found in 3.1% (3/96) of patients, and 19q-loss/1p-retain was found in 6.3% (6/96). In the 3D-radiomics signature, 2.1% (2/96) of the patients were recognized as having a 1p/19q co-deletion, and 7.3% (7/96) were classified as 1p/19q intact, suggesting that tumors with a single 1p or 19q deletion have more similarity with 1p and 19q intact tumors radiologically. Nevertheless, the underlying mechanism of single 1p or 19q loss, as well as the diversity in clinical and radiological influences, remains to be further investigated.

This study has a few limitations. First, this was a single center investigation with limited sample size to ensure the homogeneity of imaging data to perform re-sampling. Further large-scale, multicenter studies may be essential in improving the prediction performance as well as validating the radiomics signature. Second, only CE-T1-weighted and T2-weighted images were included since they are more widely applied in clinical practice. Alternative MRI modalities (e.g., perfusion-weighted imaging, diffusion-weighted imaging, magnetic resonance spectroscopy) and other imaging techniques (e.g., positron emission tomography) may also contribute to the non-invasive measurement of 1p/19q co-deletion status, and should be further investigated or combined to structural-based MRI modalities (46–48). Third, treatment strategies and patient prognosis were not integrated in the current study since the majority of the patients did not meet their endpoint. Comprehensive data with long-term follow-up may be intensively needed to prove the clinical significance of the radiomics signature. Finally, the biological processes underlying the radiomics signature remain to be investigated.

In conclusion, conventional MRI-based radiomics signature can differentiate 1p/19q co-deletion status in WHO grade II and III gliomas regardless of the predetermined IDH status. 3D-CE-T1-weighted image-derived radiomics features are more favorable than SC-CE-T1-weighted features in the establishment of radiomics signatures.

## Data Availability Statement

The raw data supporting the conclusions of this article will be made available by the authors, without undue reservation.

## Ethics Statement

The studies involving human participants were reviewed and approved by Institutional Review Board of Peking Union Medical College Hospital. The patients/participants provided their written informed consent to participate in this study.

## Author Contributions

HY, YW, WM, and FF designed the study. ZK, YZ, SL, ZL, YL, and DZ collected the data. ZK, CJ, DL, WC, PL, TY, YW, and FF analyzed and interpreted the data. ZK, CJ, YZ, SL, DL, ZL, WC, PL, TY, and YL drafted the work. DZ, HY, YW, WM, and FF substantively revised the work. All authors approved the final version of manuscript and submission to the journal.

## Conflict of Interest

The authors declare that the research was conducted in the absence of any commercial or financial relationships that could be construed as a potential conflict of interest.

## Abbreviations

3D-CE-T1-weighted: three-dimensional contrast enhanced T1-weighted
AUC: area under curve
CNS: central nervous system
GLCM: gray level co-occurrence matrix
GLDM: gray level dependence matrix
FISH: fluorescence in situ hybridization
FLAIR: fluid-attenuated inversion recovery
GTR: gross total resection
LASSO: least absolute shrinkage and selection operator
LGG: lower grade glioma
MRI: magnetic resonance imaging
PCR: polymerase chain reaction
ROC: receiver operating characteristic
ROI: region of interest
SC-CE-T1-weighted: simulated-conventional contrast-enhanced T1-weighted
SD: standard deviation
STR: subtotal resection
SVM: support vector machine
WHO: World Health Organization.
