# Supplementary material for: Thin-Slice Magnetic Resonance Imaging-Based Radiomics Signature Predicts Chromosomal 1p/19q Co-deletion Status in Grade II and III Gliomas
Source: Front Neurol. 2020 Oct 22;11:551771. doi: 10.3389/fneur.2020.551771 (PMC7642873; doi:10.3389/fneur.2020.551771)
Supplement: Supplementary file 2 [file Data_Sheet_2.DOCX]

**Supplementary Material 2. The Selected Features in the SC-Radiomics Signature**

| **Feature Name** | **Modality** | **Matrix** |
| --- | --- | --- |
| Small Area Low Gray Level Emphasis | SC-CE-T1-weighted | GLSZM |
| Correlation | T2-weighted | GLCM |
| Inverse Difference Moment Normalized | T2-weighted | GLCM |
| Inverse Variance | T2-weighted | GLCM |

Abbreviations: SC-CE-T1-weighted, simulated-conventional contrast enhanced T1-weighted; GLCM, Gray level co-occurrence matrix; GLSZM, Gray level size zone matrix.
