# Supplementary material for: Thin-Slice Magnetic Resonance Imaging-Based Radiomics Signature Predicts Chromosomal 1p/19q Co-deletion Status in Grade II and III Gliomas
Source: Front Neurol. 2020 Oct 22;11:551771. doi: 10.3389/fneur.2020.551771 (PMC7642873; doi:10.3389/fneur.2020.551771)
Supplement: Supplementary file 3 [file Data_Sheet_3.DOCX]

**Supplementary Material 3. Bar Charts of the 3D-Radiomics Signature**

| 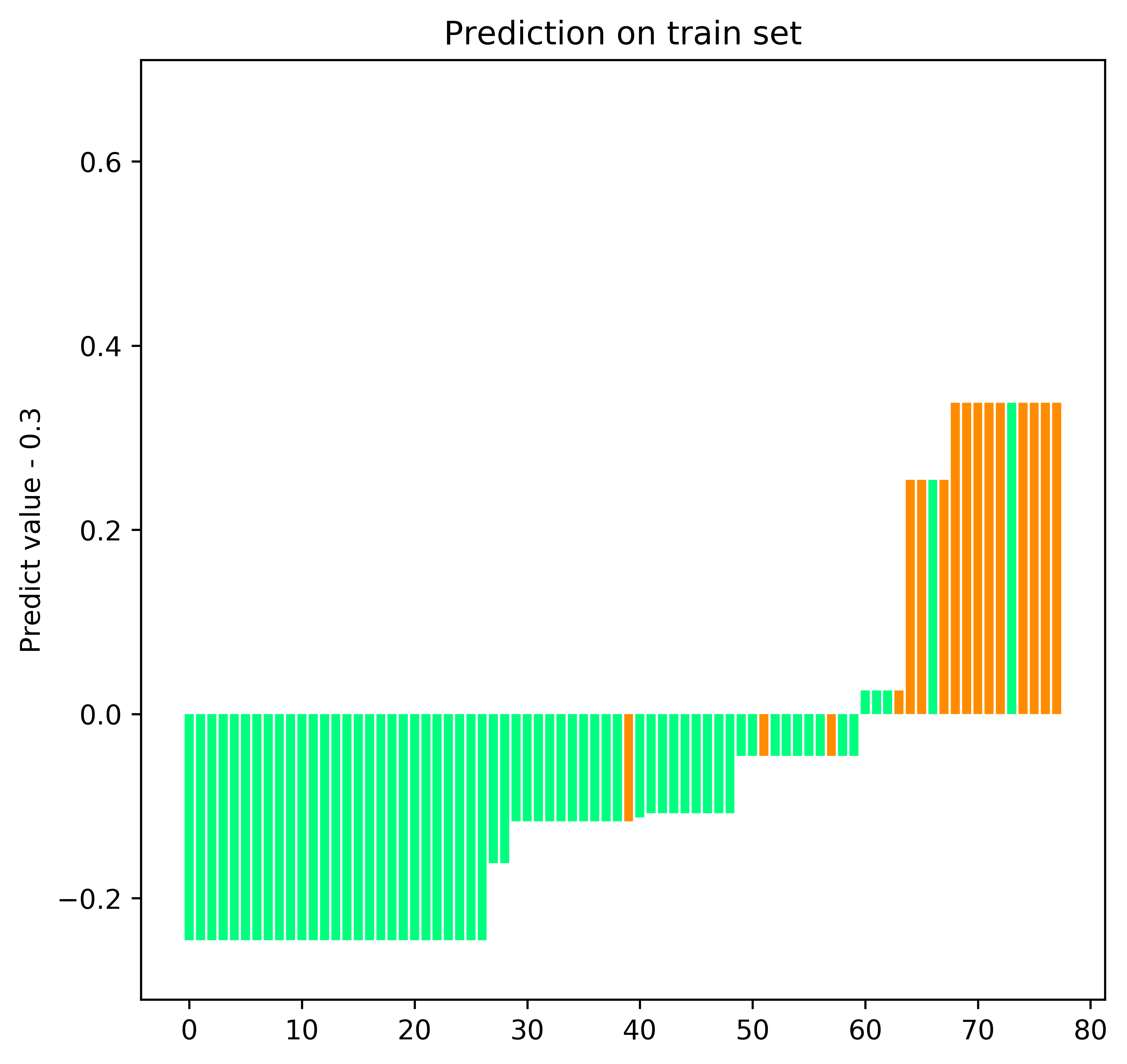 | 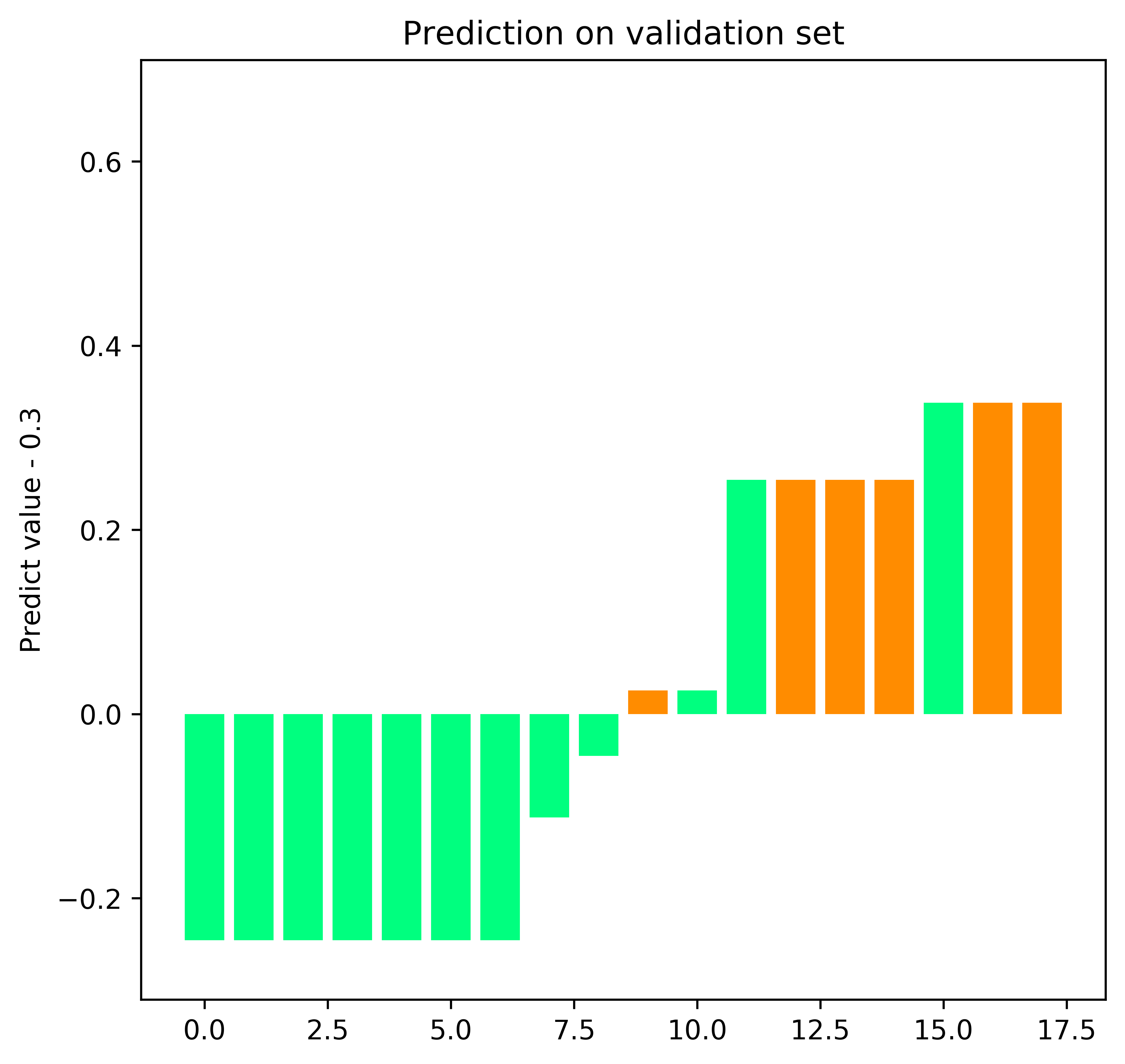 |
| --- | --- |
| Bar charts was applied to display the performance of 3D-radiomics signature in the training dataset (left) and validation dataset (right). Orange bars with prediction value minus threshold (0.3) > 0 and green bars with prediction value minus threshold (0.3) < 0 suggest accurate classification of the radiomics signature, and vice versa. | |
